# Transcription factor hubs exhibit gene-specific properties that tune expression

**DOI:** 10.1101/2025.04.07.647578

**Published:** 2025-04-08

**Authors:** Samantha Fallacaro, Apratim Mukherjee, Meghan A. Turner, Hernan G. Garcia, Mustafa Mir

**Affiliations:** 1 Department of Cell and Developmental Biology, Perelman School of Medicine, University of Pennsylvania; Philadelphia, PA 19104, USA; 2 Developmental, Stem Cell, and Regenerative Biology Graduate Group, Perelman School of Medicine; Philadelphia, PA 19104, USA; 3 Center for Computational and Genomic Medicine, Children’s Hospital of Philadelphia; Philadelphia, PA 19104, USA; 4 Biophysics Graduate Group, University of California at Berkeley, Berkeley, USA; 5 Current Address: Allen Institute for Brain Science, 615 Westlake Ave N, Seattle, WA, USA 98109; 6 Department of Molecular and Cell Biology, University of California, Berkeley, CA, USA; 7 Department of Physics, University of California, Berkeley, CA, USA; 8 Institute for Quantitative Biosciences-QB3, University of California, Berkeley, CA, USA; 9 Chan Zuckerberg Biohub – San Francisco, San Francisco, CA, USA; 10 Howard Hughes Medical Institute, Children’s Hospital of Philadelphia; Philadelphia, PA 19104, USA; 11 Epigenetics Institute, University of Pennsylvania Perelman School of Medicine; Philadelphia, PA 19104, USA

## Abstract

The spatial and temporal control of gene expression relies on transcription factors binding to and occupying their target sites. Transcription factor hubs—localized, high-concentration microenvironments—promote transcription by facilitating binding and recruitment of transcriptional machinery and co-factors. Hubs are often thought to have emergent nucleus-wide properties depending on transcription factor nuclear concentrations and intrinsic, protein sequence-dependent properties. This global model does not account for gene-specific hub regulation. Using high-resolution lattice light-sheet microscopy in *Drosophila* embryos, we examined hubs formed by the morphogen transcription factor, Dorsal, at reporter genes with distinct enhancer compositions. We found that *snail* recruits long-lived, high-intensity hubs; *sog* exhibits shorter-lived, lower-intensity hubs; and *hunchback*, lacking Dorsal binding sites, shows only transient hub interactions. Hub intensity and interaction duration correlate with burst amplitude, RNAPII loading rate, and transcriptional output. These findings challenge the global view of hub formation and support a model where hub properties are locally tuned in a gene-specific manner to regulate transcriptional kinetics.

## Introduction

The spatial and temporal regulation of gene expression during embryonic development depends on the ability of sequence-specific transcription factors to find and bind their target sites within the crowded nuclear environment. Live-imaging and single-molecule tracking experiments have shown that transcription factors bind their genomic targets transiently with residence times on the order of tens of seconds, and these factors accumulate around target genes driving an increase in their binding frequency (1–6). These high-local concentration clusters, or hubs, catalyze the recruitment of transcriptional machinery and amplify gene activation (7–20). These clusters are referred to as condensates when they exhibit properties consistent with formation via liquid-liquid phase separation mechanisms (21–23). Under the phase separation model, transcriptional condensate formation is dependent on the nuclear concentrations of their constituents and intrinsic sequence-specific properties of the proteins, such as the composition of intrinsically disordered regions (IDRs) that promote multi-valent homo- and heterotypic interactions (22). The biophysical properties of hubs are thus often treated as protein-specific and uniform across the nucleus, and how these properties may vary depending on their genomic localization is not often considered.

Hub localization to genomic sites is dynamically influenced by a combination of targeting through a transcription factor’s protein-protein and protein-DNA interactions, the affinity for the target site, and the presence of specific cofactors (10, 15, 19, 24–28). Modulating any one of these components to hub localization has functional consequences. A transcription factor’s local network of interaction partners also plays a role in generating compositionally and functionally distinct hubs (25, 28, 29). Modulation of binding sites has a direct impact on the amount of a factor present at a locus and on the transcriptional output in a concentration-dependent manner (10, 30). Recent work has suggested a spatio-temporal relationship where the proximity of a hub to its regulatory elements can increase transcriptional bursting (15, 31–33). If hubs directly influence transcription factor binding and transcriptional output, which is differentially regulated at different genes, their properties should be highly gene-dependent rather than globally dictated by transcription factor identity and nuclear concentration.

To investigate how transcription factor hub properties differ to regulate transcription in a gene-specific context, we leverage the *Drosophila* dorsoventral patterning system. In this system the transcription factor Dorsal forms a nuclear concentration gradient and activates distinct sets of genes in a spatially dependent manner along the embryo’s dorsoventral axis (34–36). Due to its morphogenic nature, Dorsal-regulated gene expression provides a natural framework to determine whether hubs function uniformly or differentially at multiple target genes and at varying concentrations. Previous work in this system has shown that transcription factor hubs containing Bicoid, Zelda, or Dorsal are recruited to specific genomic loci even at low concentrations and suggest that morphogen hubs play an active role in fine-tuning transcriptional responses to spatial gradients (7, 8, 19, 30, 37, 38). Here, we use high-resolution lattice light-sheet imaging to analyze how Dorsal hubs interact with transcriptionally active loci along its concentration gradient and how hubs are related to transcriptional bursting kinetics. We demonstrate that Dorsal hubs do not behave as uniform accumulations but instead exhibit gene-specific regulatory properties. Hubs rapidly form after mitosis and are maintained throughout interphase, yet their intensity, stability, and interactions are specific and tunable to target genes. The presence of a hub increases transcriptional activity, and hub intensities and interaction durations are highly correlated with burst amplitude, loading rate, and output. These findings suggest that hub properties and function are regulated in a gene-specific manner.

## Results

### Hubs form immediately after Dorsal nuclear import in ventral and lateral surface nuclei

**Fig. 1.**
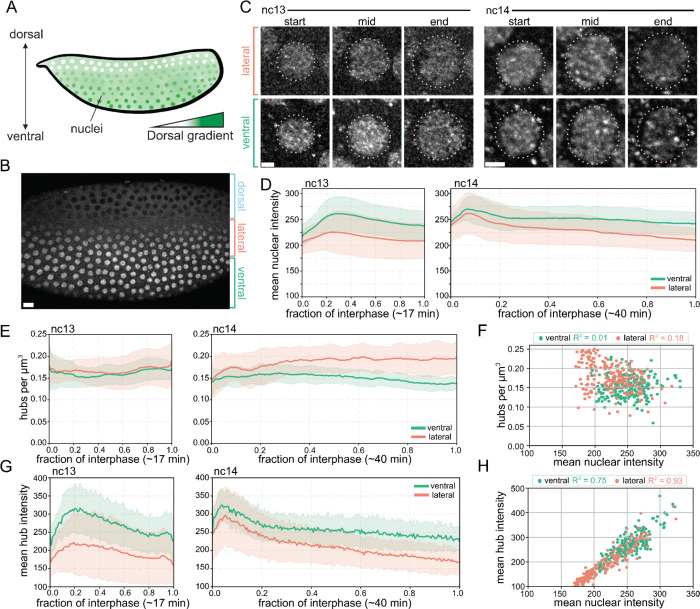
Nuclear Dorsal hub densities are constant in interphase but hub enrichment depends on Dorsal nuclear concentrations. **(A)** Schematic of the Dorsal gradient in *Drosophila* embryos. Dorsal translocates into ventral surface nuclei at higher rates creating a nuclear concentration gradient along the dorsoventral axis. **(B)** Maximum intensity projection image showing nuclear gradient of Dorsal-mNeonGreen in a *Drosophila* embryo in nc13. Scale bar is 15 microns. **(C)** Images of a single z-slice of Dorsal-mNeonGreen in the nucleus of an embryo at the start, middle, or end of nc13 (left) and nc14 (right). Scale bars are 2 microns. **(D)** Average nuclear mean intensity in ventral (green) or lateral (orange) nuclei. Shading shows standard deviation between embryo replicates. N = 5 nuclei per embryo, 3 embryo replicates for each axis position. **(E)** Average hub density (number of hubs per cubic micron) in ventral (green) or lateral (orange) nuclei. Shading shows standard deviation between embryo replicates. **(F)** Scatter plot of hub densities vs. mean nuclear intensities. R^2^ value is Pearson’s correlation (N = 5814 ventral hubs, 6928 lateral hubs from N = 3 embryos per axis position). **(G)** Mean intensities of Dorsal hubs in ventral (green) or lateral (orange) nuclei. Solid line shows average intensity and shading shows standard deviation between embryo replicates. **(H)** Scatter plot showing hub mean intensity and mean nuclear intensity. R^2^ is Pearson’s correlation.

To investigate the biophysical properties of Dorsal hubs we performed lattice light-sheet imaging of blastoderm stage *Drosophila* embryos expressing endogenously tagged Dorsal-mNeonGreen in the 13th and 14th syncytial nuclear cycles (nc13 and nc14). Dorsal forms a nuclear concentration gradient where Dorsal is highly concentrated in nuclei at the ventral surface, has a moderate-low concentration in lateral nuclei, and is excluded from the nuclei on the dorsal surface (Figure 1A). The nuclear concentration gradient is re-established after each nuclear division. At the onset of mitosis the cytoplasmic-nuclear cocentration ratio is flattened, after nuclear membrane reformation the gradient is re-established through a balance of nuclear import and export during interphase (36). We observed that Dorsal hubs reform immediately following telophase in each nuclear division (Movie 1). Hubs are then visible throughout interphase and are present in nuclei across both the ventral and lateral surfaces (Figure 1B–C). Interestingly, at low Dorsal concentrations–particularly in lateral nuclei at and near the dorsal-lateral transition point–hubs form even as the nucleoplasmic Dorsal concentration is diminished (Figure S1A,B, Movie 2). This is consistent with previous literature indicating the presence of hubs along entire concentration gradients despite diminishing nuclear concentrations (7, 8, 30, 37).

To understand how nuclear concentrations influence hub formation, we compared ventral nuclei along the presumptive ventral furrow and lateral nuclei at the dorsal-lateral transition point (Figure S1A,B). Despite ventral nuclei having a higher concentration of Dorsal than lateral nuclei, both the ventral and lateral Dorsal nuclear intensity rise and then fall during interphase (Figure 1D). To compare Dorsal hubs in these nuclei, we developed a custom analysis pipeline to detect hubs and quantify their intensities, locations, and stabilities (Figure S1C, Movie 3). Despite the change in nuclear concentration during interphase, the density of Dorsal hubs (number of hubs per volume) remains largely constant during the interphases of nc13 and nc14 (Figure 1E–F, S1D–G). Although the density of hubs is uncoupled with nuclear concentration, the average hub intensity is strongly correlated with nuclear Dorsal concentration (Figure 1G–H, S1H). Taken together, these data suggest that hubs may form rapidly at specific sites immediately following mitosis and then incorporate increasing amounts of Dorsal as nuclear concentrations increase.

### Dorsal hubs preferentially interact with target sites

**Fig. 2.**
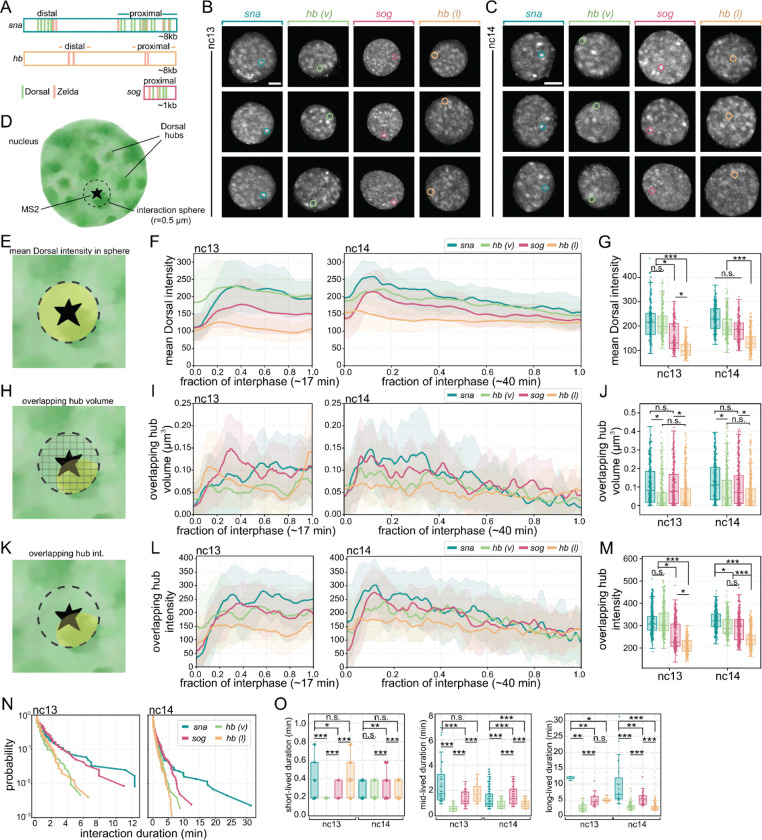
Dorsal hubs are stably retained at target genes and show transient interactions at non-targets. **(A)** Schematic depicting enhancers with Dorsal and Zelda (one cofactor that is common to all three genes) binding sites for each reporter used in this study. Each enhancer was positioned just upstream of its promoter, 24 MS2 stem loops, and the *yellow* coding sequence. Dorsal target genes, *snail* and *sog,* are expressed along the ventral and lateral surfaces respectively while *hunchback* is expressed anteriorly. *hunchback* is not a Dorsal target gene and is used as a negative control for both the anterior-ventral surface (*hb(v)*) to compare to *snail* or on the anterior-lateral surface (*hb(l)*) to compare to *sog*. **(B, C)** Representative maximum intensity projections of single nuclei with Dorsal-mNeonGreen in nc13 **(B)** and nc14 **(C)**. The MS2 reporter location is circled in each image. The cytoplasmic Dorsal signal is masked and contrast was adjusted independently to aid visualization. Scale bars are 2 microns. **(D)** A single nucleus with Dorsal hubs is depicted below. The MS2 reporter location is denoted as a star, a 0.5 micron radius sphere is considered around the MS2 spot for quantification of Dorsal signal at a gene. **(E)** Schematic showing the mean Dorsal intensity in the interaction sphere around the MS2 spot. **(F)** Line plots showing the average of mean Dorsal intensities at each gene in nc13 (left) and nc14 (right). **(G)** Boxplots showing the distribution of mean Dorsal intensities in the middle of interphase at each gene. **(H)** Schematic showing the volume of Dorsal hubs overlapping the interaction sphere around the MS2 center. **(I)** Line plots showing the average volume of Dorsal hubs overlapping at each gene in nc13 (left) and nc14 (right). **(J)** Boxplots showing the distribution of Dorsal hub volumes overlapping in the middle of interphase for each gene. **(K)** Schematic showing the mean intensity of Dorsal hubs overlapping in the interaction sphere around the MS2 center. **(L)** Line plots showing the average intensities of Dorsal hubs overlapping at each gene in nc13 (left) and nc14 (right). **(M)** Boxplots showing the distribution of the overlapping Dorsal hub intensities in the middle of interphase for each gene. **(N)** Survival plots showing the length of time any hub is consistently present within the interaction sphere for each gene for nc13 (left) and nc14 (right). **(O)** Box plots showing the average duration of consistent hub interactions within the short-, mid-, and long-lived populations determined by gaussian-mixture modelling (Figure S2D) for each gene. N = 5 nuclei per embryo and 3 embryos replicates for each gene. A Mann-Whitney U-test was performed to determine significance and following p-values were used: *p<0.05, **p<0.01, and ***p<0.001.

To determine whether Dorsal hubs preferentially form at transcriptionally active target sites, we used two-color imaging of Dorsal in conjunction with the MS2/MCP system to visualize nascent transcription. We used reporters of *snail* (*sna*) and *short gastrulation* (*sog*), known Dorsal target genes at the ventral and lateral domains respectively, as well as *hunchback* (*hb*), a non-target control in ventral (*hb(v)*) and lateral (*hb(l)*) nuclei (Figure 2A–C, S2, Movie 4, 5). Visually, we found that Dorsal hubs appear most stably associated with *sna* and slightly less so at *sog* compared to random interactions at *hb(v)* and *hb(l)* (Movie 4, 5). To quantify these hub-locus interactions, we built a custom analysis pipeline to localize and track actively transcribing loci and their interactions with Dorsal hubs (Figure 2D, Movie 6). To characterize hub interactions, we quantified (i) overall mean intensity, (ii) overlapping volume of hubs, and (iii) overlapping hub intensity of Dorsal within a 0.5 micron-radius interaction sphere around the MS2 spot (Figure 2E, H, K).

When comparing genes at the lateral surface, *sog* consistently has higher Dorsal intensity, hub volume, and hub intensity near the locus as opposed to its non-target control, *hb(l)* (Figure 2E–M). Differences on the ventral surface between *sna* and *hb(v)* are minimal in terms of intensity, yet *sna* still has a significantly increased hub interaction volume around the locus. The similarity in intensity between targets (*sna* and *sog*) and non-targets (*hb(v)* and *hb(l)*) may be in part due to higher nuclear background intensities in the ventral versus lateral domains. Furthermore, mirroring its global trend (Figure 1G–H), hub intensities at target sites fluctuate in synchrony with nuclear Dorsal intensities (Figure 2F, S3A–C), suggesting that hubs may be formed and retained at target genes.

We next quantified gene-hub interaction durations by assessing how long a hub is consistently present within the interaction sphere at each locus. We found a striking increase in the interaction durations at target genes compared to non-targets (Figure 2N). We classified hub interactions using a three-component Gaussian mixture model into short-, mid-, and long-lived interactions (Figure S3D). We found that the durations of mid- and long-live hub contacts in target genes are significantly increased compared to non-targets (Figure 2O, S3D): *sna* has the longest persistent interactions (1.35±0.83 minutes mid- and 10.04±7.19 minutes long-lived in nc14) followed by *sog* (1.55±0.73 minutes mid- and 5.39±1.98 minutes long-lived in nc14). Meanwhile, the non-targets have shorter interaction durations at both the ventral and lateral surfaces (*hb(v)* mid: 0.90±0.31 minutes; long: 3.00±1.33 minutes, and *hb(l)* mid: 0.88±0.32 minutes; long: 2.77±1.09 minutes in nc14). These results suggest that while Dorsal hubs can transiently interact with non-target loci, stable hub retention is restricted to target genes. This next prompted us to understand how hub interaction kinetics correlate with transcriptional activity at target and non-target genes.

### Dorsal hub persistence and intensity correlate with transcriptional activity at target genes

**Fig. 3.**
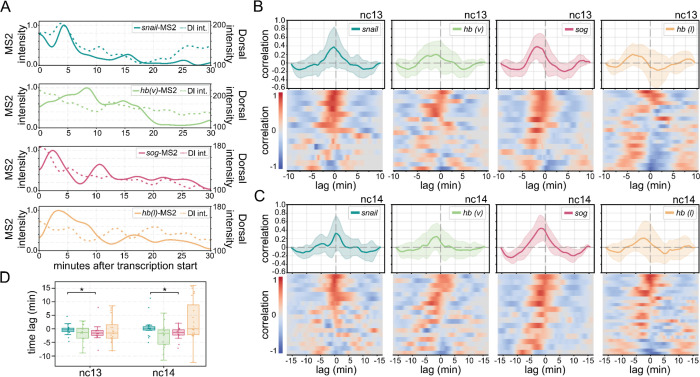
Dorsal hub dynamics correlate with transcriptional activity. **(A)** Representative traces of MS2 intensity and Dorsal intensity at each reporter gene in nc14. **(B, C)** Cross-correlations of normalized MS2 intensity and Dorsal intensity for each gene in nc13 and nc14. Heat maps show the cross-correlation for individual nuclei and the corresponding average cross-correlation is shown above each heatmap, shaded regions depict standard deviation. **(D)** Box plot shows the distribution of the time lag with the highest correlation. Mann-Whitney U-test was performed to determine significance and following p-values were used: *p<0.05, **p<0.01, and ***p<0.001.

To assess whether Dorsal hub interactions directly correlate with transcription, we analysed the relationship between hub presence, transcriptional kinetics, and nuclear Dorsal intensity (Figure 3A, S4A). We found that Dorsal intensity and hub contacts are uncorrelated with the start of detectable transcriptional activity for non-targets, and Dorsal hubs exhibit higher intensities and contacts at *sna* and *sog* (Figure S4B). Cross-correlation analysis of normalized MS2 intensities, a proxy for time-resolved nascent transcriptional output, and Dorsal hub intensities shows that Dorsal hub intensities are strongly correlated with target gene activity, reinforcing the idea that transcriptional activation coincides with local hub enrichment in an instantaneous manner (Figure 3B–D). Interestingly, *hb* transcription in both ventral and lateral nuclei is not completely uncorrelated with Dorsal hub interactions, however, there is a greater variation among nuclear replicates than observed for target genes (Figure 3D).

Notably, differences emerge in the time-lag of peak intensity correlations between *sna* and *sog*. To understand the time difference between the signal peaks, we quantified the average time lag with the highest correlation. We found that hubs at *sna* display an average time lag of −0.32±2.17 minutes in nc13 and 1.06±4.94 minutes in nc14 with nascent transcriptional activity, whereas hubs at *sog* have a time delay of −1.22±3.21 minutes in nc13 and −1.81±2.46 minutes in nc14 minutes with the MS2 signal (Figure 3B–D). This suggests that while both genes recruit Dorsal hubs during activation, the nature of their interactions varies. The most likely explanation for this is the stability of hubs at the *sna* locus which are persistently visible (Movie 4, 5). We suspect these differences could arise from the affinity of *cis*-regulatory elements for Dorsal and its cofactors.

### Dorsal hub dynamics predict transcriptional output and burst parameters

**Fig. 4.**
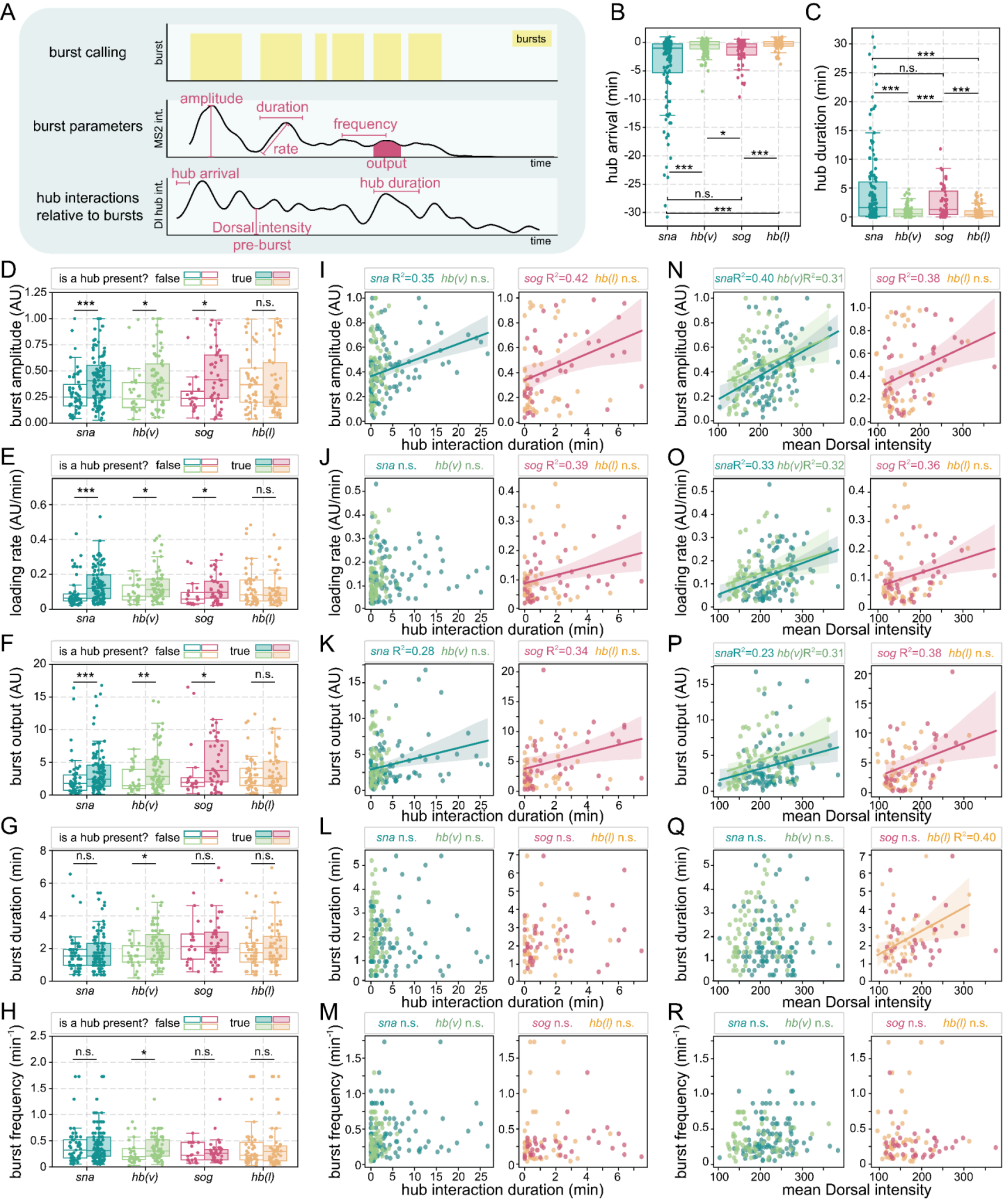
Dorsal hub presence modulates transcriptional transcriptional burst parameters. **(A)** Schematic showing the burst calling, burst parameters extracted from the MS2 intensity, and the hub interaction parameters quantified from the Dorsal hub overlap and intensity. Bursts are identified in yellow. **(B)** Box plot showing the hub arrival time (first time point when there is a hub within 0.5 micron radius of the MS2 spot) relative to the start of a burst. For each boxplot, a Mann-Whitney U-test was performed to determine significance and following p-values were used: *p<0.05, **p<0.01, and ***p<0.001. **(C)** Box plot shows the duration in minutes that a hub is still in proximity after the start of a burst. **(D-E)** Boxplots showing each quantified burst parameter (burst amplitude **(D),** loading rate **(E)**, burst output **(F)**, burst duration **(G)**, and burst frequency **(H)**) if a high intensity hub is present at the start of the burst or not. **(I-M)** Scatter plots showing each burst parameter with the hub interaction duration after a burst **(I-M)** and mean Dorsal intensity before the burst **(N-R)** only when a high intensity hub is present at the start of the burst for ventrally imaged genes (left) and laterally imaged genes (right). A Pearson’s correlation R^2^ and linear regression is shown when p>0.05 for the correlation.

We next examined whether specific hub properties are correlated with transcriptional burst kinetics. We first segmented MS2 traces into individual bursts using the rate of change of MS2 intensities (Figure 4A, S5A–D). For each burst, we identified the time when a hub first arrives at the locus and how long after the start of the burst the hub is retained at the locus. As visually evident, Dorsal hubs at *sna* exhibit much greater stability than *sog* during periods of active transcription (Figure 4B–C, Movie 4, 5). We found that hubs at target genes often arrive before a burst and dwell after the burst start at the locus for a longer amount of time compared to non-targets. Dorsal hubs at *sna* are present on average 20.95 minutes before non-first bursts in a sequence and remain on average for 23.16 minutes after each the start of each burst compared to *hb(v)* where hubs arrive on average 4.15 minutes before bursts and remain on average 4.99 after the burst. At the lateral surface hubs at *sog* arrive 8.38 minutes before on average and remain 13.02 minutes after the burst compared to hubs at *hb(l)* arriving 1.48 minutes before and remaining for 4.40 minutes on average after each burst. This further suggests specificity and tunability of hub interactions at genes.

We next assessed how burst parameters might be affected by the presence of a hub just before the start of the burst. We first examined the presence of any hub regardless of its intensity at the start of a burst, and found that with any hub present, both ventral genes have increased burst amplitude, loading rates, and burst outputs (Figure S5E–G). Interestingly, *sna* burst duration and frequency was not changed in the presence of a hub while *hb(v)* was. We saw no significant differences for burst parameters for either of the lateral genes. Then, we tested how changes in hub interaction duration length and intensity tune transcriptional kinetics (Figure S5J–S). We found that hub durations positively correlate with *sna*’s burst amplitude and output while Dorsal intensity positively correlates with *sna*, *hb(v)*, and *sog* amplitude, loading rate, and output but not duration or frequency.

Seeing how *sog* is impacted by changes in Dorsal intensity, we next decided to see how burst parameters are impacted by high intensity hubs using an intensity threshold (set to the average intensity of a ventral hub) to define hub interactions. We found that now in addition to the ventral genes, *sog* has a significant increase in amplitude, loading rate, and output when a high intensity hub is present (Figure 4D–H). Additionally, these three parameters are positively correlated with both Dorsal intensity and hub interaction durations (Figure 4I–R).

Taken together, the relationship between hub duration and burst output indicates that stable hub interactions may facilitate increased transcriptional activity through changing amplitude, loading rate, and output but not duration or frequency. By fine-tuning hub properties like concentration and stability at target genes, hubs may refine transcriptional activity in a locus-specific manner. Interestingly, transcriptional output at hb on the ventral surface is impacted by the presence of a Dorsal hub which is likely not an indicator of a direct role of Dorsal in regulating this locus but rather that Dorsal hubs are likely multifactorial environments containing other transcriptional regulators that can impact non-target gene expression when in proximity. These findings suggest that Dorsal hubs act as dynamic yet functionally distinct transcriptional regulators at different genes, reinforcing their importance in coordinating transcriptional precision across axes.

## Discussion

Our findings demonstrate that transcription factor hubs are not uniform entities but instead exhibit gene-specific regulatory properties. We show that Dorsal hubs display differential stability at distinct loci, indicating that the biophysical properties of hubs are not dictated solely by global properties such as nuclear concentrations (Figure 2). Instead, we observe that some genes recruit stable, long-lived Dorsal hubs while others recruit Dorsal transiently, suggesting that the properties of hubs are tuned by local regulatory elements and chromatin features. This observation supports the growing view that transcription factor hubs do not behave as phase-separated condensates but instead form through cooperative interactions with enhancers and binding partners that determine their stability and function (18, 19, 25, 27, 28, 33, 37, 39).

**Fig. 5.**
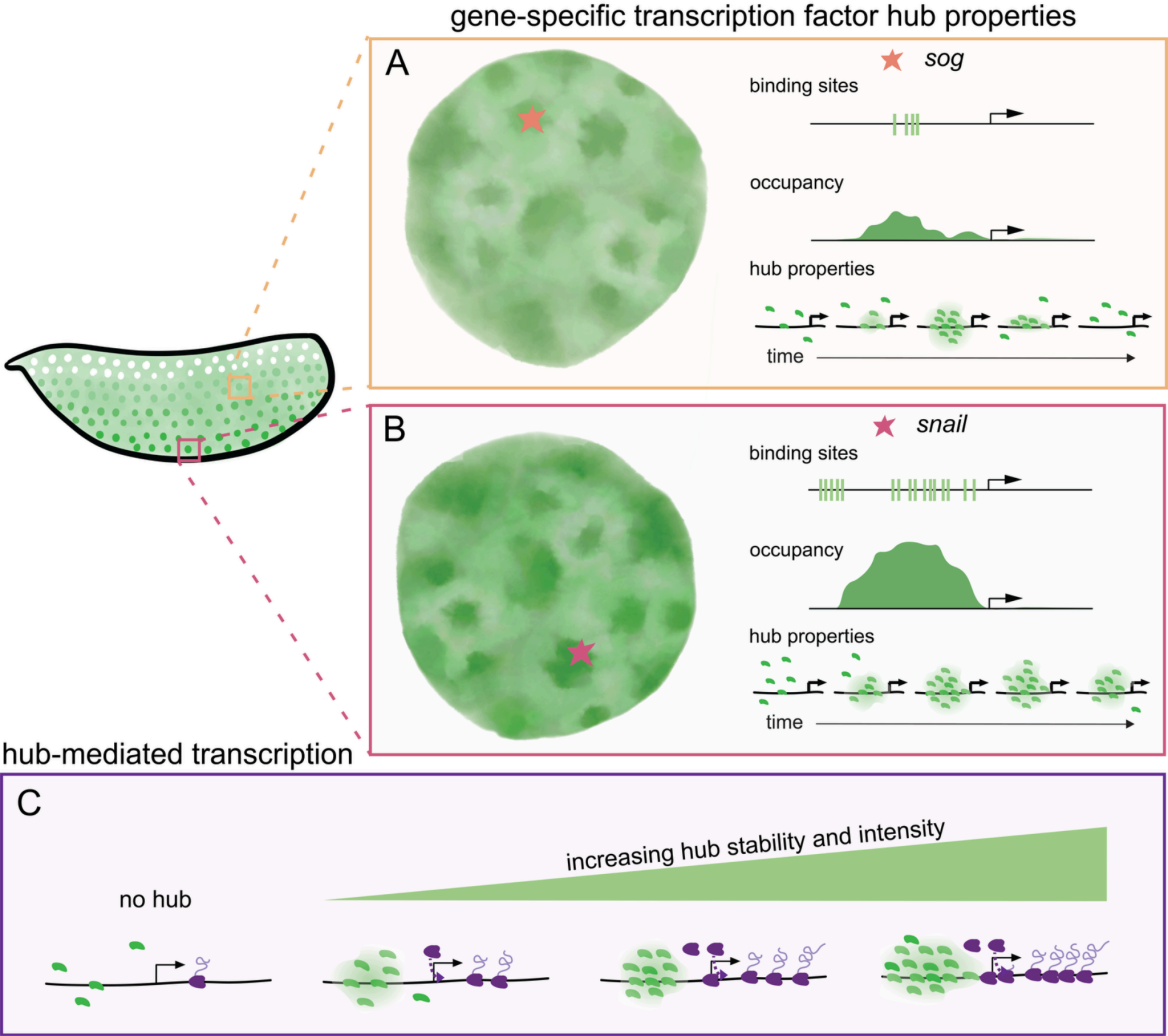
Gene-specific transcription factor hub properties mediate transcriptional kinetics. **(A, B)** Binding sites at *cis-*regulatory regions mediate changes in Dorsal gene occupancy through dynamic hub formation. As a result, different sites such as *sog* **(A)**, and *snail* **(B)** have differing concentrations and stability of Dorsal hubs. **(C)** Hubs may fine-tune transcriptional kinetics by recruiting more transcriptional machinery when hubs are more stable and concentrated.

We propose a model in which enhancers encode the properties of transcription factor hubs, while dynamic hub interactions ultimately dictate gene occupancy and transcriptional kinetics (Figure 5). The differences in Dorsal binding sites at each of our MS2 reporters (Figure 2) may correlate with hub dynamics: the *snail* enhancer contains more Dorsal binding sites, the longest hub interactions and the highest intensity hubs, *sog* has less Dorsal binding sites and shorter-lived hubs with lower intensity, and *hunchback* with no Dorsal binding sites exhibits only transient interactions with Dorsal hubs regardless of its dorsoventral position. This also matches the relative Dorsal occupancy in each of these genes’ endogenous enhancer regions (Figure S2), suggesting that transcription factor motif combinations play a role in hub formation (40). Interestingly, despite lacking Dorsal binding sites, in ventral regions higher-intensity Dorsal hubs still transiently interact with *hunchback* (Figure 2), and these interactions can lead to significant transcriptional enhancement (Figure 4). This nonspecific hub interaction leading to a boost in transcriptional activity suggests that transcription factor hubs, which are likely enriched with cofactors and transcriptional machinery, boost gene activation independent of direct binding (9, 12, 27, 41). However, this Dorsal hub-mediated enhancement of *hunchback* transcription is not observed in lateral regions, likely due to the lower probability of high-intensity hubs in these regions.

*Cis*-regulatory element control of transcription factor hubs is not completely unprecedented. Prior work demonstrated that removing Zelda binding sites at *sog* reduced both Dorsal clustering and transcription, particularly in lateral regions where Dorsal concentration is lower (30). Other work also demonstrated that high-affinity Bicoid binding sites did not result in increased Bicoid clustering intensity, suggesting that clustering is not simply dictated by affinity (37). However, this study did not account for differences in number of binding sites or hub interaction times, which may be a crucial distinguishing factor, akin to the differences observed between *snail* and *sog* in our data. Overall, this suggests that enhancer grammar—including binding site affinity, composition, and arrangement—may play a key role in determining hub properties and function. Future work may involve targeted enhancer dissections to better understand how these sequence features influence hub formation.

Our findings reveal a strong relationship between hub dynamics and transcriptional activity, with burst amplitude, loading rate, and total output significantly increasing when hub interaction duration and intensity are higher (Figure 4). This aligns with previous work demonstrating that increasing transcription factor clustering leads to larger burst sizes, although this was achieved by altering protein structure through the addition of polyQ repeats to Bicoid (10). In contrast, studies on Gal4 clusters found no difference in burst size when the clusters were positioned closer to their target site (32), whereas our results show that Dorsal hubs increase burst size when closer to their targets. Additionally, *in vivo* single molecule tracking of Gal4 at its target site suggests that burst duration and size are primarily determined by transcription factor dwell time and binding affinity (32). Since Dorsal hub presence did not alter burst duration, it is likely that hubs do not impact Dorsal residence time. Instead, these findings suggest that hubs may influence transcription through stabilizing interactions with or increasing the recruitment of transcriptional machinery (27, 33). Transcriptional machinery such as RNA polymerase II (RNAPII) has also been shown to form dynamic clusters that correlate closely with transcriptional bursts and likely contain a mixture of initiating and elongating complexes (18). Whether transcription factor hubs actively engage with RNAPII clusters to promote gene activation—or merely arise as a consequence of multiple molecules independently accumulating at active loci—remains an open question for future study.

Despite inadequate evidence to distinguish hub formation from liquid-liquid phase separation and other mechanisms of formation, the prevailing phase separation model suggests that transcription factor hubs form through generic multivalent interactions driven by intrinsically disordered regions (IDRs) (21, 22, 42). This implies homogeneous, compositionally similar condensates across the nucleus (22), which is also inconsistent with our observations of gene-specific Dorsal hubs and the growing viewpoint that differential heterotypic protein interactions may have consequences for localization and composition (25, 29). Recent work suggests that there is a “Goldilocks zone” of hub behavior—where hubs that are too weak or transient fail to activate transcription, and overly stable or aggregated hubs may impede dynamic regulatory processes (39, 43). Our data support this nuanced view: Dorsal hubs exhibit distinct lifetimes and intensities depending on their genomic target, and these features are tightly correlated with transcriptional output. Future work should be done to test the constituency of these hubs and whether or not they are distinct at different target genes.

Together, we show that Dorsal transcription factor hubs exhibit gene-specific regulatory properties that dictate genomic occupancy and transcriptional output. We propose a model in which enhancers encode hub properties, while hub interactions dictate gene occupancy, ultimately shaping transcriptional kinetics through burst amplitude, loading rate, and total output. These findings challenge the current view of hubs as passive transcriptional amplifiers and instead support a model in which hubs are dynamically tuned to meet the regulatory demands of individual genes. By integrating high-resolution imaging with enhancer dissection and transcription factor kinetic measurements, future studies can further clarify how hubs orchestrate gene-specific transcriptional regulation within the nucleus.

## Materials and Methods

### Embryo Preparation for Live Imaging

For each experiment, *Drosophila* embryos were generated by crossing virgin female flies heterozygous for MCP-mCherry and Dorsal-mNeonGreen to males containing the MS2 reporter of interest. The resulting embryos were collected for live imaging. Only embryos positive for all three (Dorsal-mNeonGreen, MCP-mCherry, and MS2-gene-of-interest) were imaged.

Embryos were collected over a 90-minute window and manually dechorionated by gently rolling them on double-sided tape using a blunt needle until the chorion was removed. Dechorionated embryos were then arranged on an agar pad before being transferred onto an adhesive heptane-acrylic spot, prepared by dissolving double-sided tape in heptane on a 25 mm diameter glass coverslip.

### Microscope Calibration and Imaging

The lattice light-sheet microscope (49) used in this study is a home-built, modified implementation based on the adaptive optics-equipped lattice light-sheet (50) system originally developed by the Betzig lab at HHMI Janelia Research Campus. For all experiments, 488 nm and 589 nm laser lines were used. These laser beams were first expanded to a combined 2 mm diameter, passed through a half-wave plate to adjust polarization, and relayed into an acousto-optic tunable filter (AOTF) (Quanta-Tech, AA Opto Electronic) to selectively control wavelength and power modulation. The collimated beams were then expanded in one dimension using a Powell lens (Laserline Optics Canada), and their width was adjusted and collimated using a pair of cylindrical lenses. The beam was subsequently relayed onto a grayscale spatial light modulator (Meadowlark Optics, AVR Optics) after passing through a second half-wave plate, where it was diffracted and projected onto a custom annular mask to define the minimum and maximum numerical aperture (NA) of the light-sheet while blocking undiffracted light. The light from the annulus was demagnified and relayed onto a resonant galvanometer scanner (Cambridge Technology, Novanta Photonics), conjugate to the sample plane, to mitigate shadowing artifacts and improve uniformity by introducing a slight wobble in the excitation angle. Finally, the light was projected onto a pair of galvanometer scanning mirrors (Cambridge Technology, Novanta Photonics)conjugate to the pupil plane, allowing scanning of the light-sheet along the x and z optical axes before being focused onto the sample using a Thorlabs TL20X-MPL excitation objective. Fluorescence emission was collected orthogonally by a Zeiss 20×, 1.0 NA detection objective and relayed onto a deformable mirror (ALPAO) positioned in the detection path to correct optical aberrations, as previously described (50). The emitted signal was then split using a Semrock Di03-R561-t3–25×36 dichroic beam splitter and directed onto two sCMOS detectors (Hamamatsu ORCA Fusion). The green emission channel (for 488 nm excitation) was filtered using a Semrock FF03–525/50–25 emission filter and a Chroma ZET488NF notch filter to reject laser light, while the red emission channel (for 589 nm excitation) was filtered using a Semrock FF01–615/24 emission filter and a Chroma ZET594NF notch filter.

All imaging used a multi-bessel lattice sheet with maximum numerical aperture to minimum numerical aperture ratio of 0.4/0.3 488 nm (used to excite mNeonGreen) and 589 nm (used to excite mCherry). Two color channels were acquired sequentially for a volume of 18.9 μm sampling z-planes spaced 0.3 μm apart with an exposure time of 30 msec. A laser power of 0.124 mW was used for the 488 nm laser and 1.344 mW for the 589 nm laser. Each volume was acquired every 11.56 seconds during acquisition for a total time of ~11.56 seconds in between stacks.

### Image Analysis

All volumetric imaging datasets were pre-processed before downstream analysis using GPU-accelerated 3D deconvolution via CUDA (51). Each dataset was deconvolved using a Richardson-Lucy based algorithm with 8 iterations, utilizing point spread functions (PSFs) captured from bead images acquired on the day of imaging to ensure accurate correction. For visualization and rendering, we employed a custom Imaris converter, leveraging fast TIFF and Zarr file readers to efficiently generate Imaris files (46).

Following deconvolution, images underwent nuclear segmentation using a custom-trained model in Cellpose 2.0 (48). Ground truth training data was generated through a combination of micro-SAM (47) and manual curation on MCP-mCherry images. The trained Cellpose model was then applied to segment individual slices across each dataset. To improve segmentation accuracy, a post-processing pipeline was developed to stitch segmented slices together and interpolate missing nuclear structures. We then implemented a nearest-neighbor tracking algorithm to follow nuclei across interphase in each nuclear cycle.

To quantify Dorsal hub properties, we used a custom image analysis pipeline we previously used (18, 19). First, nuclear intensity was normalized to its mean value to assess local transcription factor enrichments above background levels. Hubs were segmented by first applying a median filter to reduce noise, followed by image erosion and reconstruction to enhance feature contrast. The reconstructed image was then subtracted from the median-filtered image to generate a binary mask identifying high-density regions. To resolve closely associated hubs, local maxima peaks were identified and used as markers for watershed-based segmentation, ensuring separation of fused or amorphous structures. Finally, region properties were extracted to quantify hub features, including integrated intensity, mean intensity, and size.

### MS2 Tracking and Burst Analysis

After segmenting nuclei and hubs, we manually select nuclei for further analysis under the criteria of checking if anomalies are present in the segmentation and to check if the MS2 spot is too far to the edge of the nucleus which may skew analysis. We then generate 4D images of cropped single nuclei. These selected nuclei are then subjected to another segmentation regime this time to label the MS2 spot. This regime uses a median filter, difference-of-gaussians filter, and then a percentile threshold to segment high intensity spots. The exact sigma values and percentile used was adjusted nucleus-to-nucleus to ensure the best possible MS2 segmentation. Then we identify the spot inside the nucleus with the highest intensities to be the likely MS2 spot. We then used a nearest neighbour algorithm to track the MS2 spot in time while also manually correcting any tracking errors in each nucleus. Due to the bursty nature of transcription, we interpolate the MS2 position in between bursts of visible MS2 spots and extrapolate the MS2 position 20 frames before the first appearance of the MS2 signal while correcting for the motion and size of the nucleus.

We then quantified Dorsal hub interactions at the MS2 spot by assessing the overlap between the hub labels and a 0.5 μm radius sphere centered at the MS2 weighted centroid coordinates as we have done previously (19). We could then monitor the duration, intensity, and volume overlap at each locus regardless of transcriptional activity. We additionally performed cross-correlations to examine the temporal relationship between transcriptional activity and Dorsal hub dynamics. Smoothed intensity traces for each gene were normalized and cross-correlated using np.correlate to quantify alignment over time.

We also called bursts to assess transcriptional activity by applying a Gaussian smoothing filter to the MS2 intensity and calculated the derivative to capture periods where the transcriptional activity peaks. We could then quantify parameters such as amplitude, duration, output, loading rate, and frequency based on these bursts. Based on the start times of these bursts we could then assess if a hub was present at the start of a burst, the arrival time of that hub overlapping with the MS2 region and then overlap intensity and volume. To assess if a hub was present we used two different metrics: we first assessed if any hub voxels overlapped with the MS2 region and then assessed if a hub was present and was over a threshold intensity value.

## Supplementary Material

Supplement 1 **Fig. S1. Nuclear segmentation and hub detection across the dorsoventral axis. (A, B)** Images showing where nuclei were selected at the lateral surface within a max projection of a full field of view **(A)** and for a single slice for each nuclei **(B)**. Nuclei labelled as lateral (for *sog* and *hunchback_l*) were imaged within 5 nuclear rows of the transition point (row 0 of nuclei where the cytoplasm and nuclear signals are indistinguishable). Each row has a varying nuclear to cytoplasmic ratio of signal yet all have hubs within the nucleus. Scale bar in B is 15 microns and 1 micron in C. **(C)** Images showing results of nuclear and hub segmentation. Left image shows a representative 3D rendering of Dorsal-mNeonGreen in ventral nuclei. Scale bar is 2 microns. Center image shows nuclear segmentation of the 3D rendering. Right image shows segmentation of Dorsal hubs after applying a custom segmentation code. **(D)** Average nuclear volume in ventral (green) or lateral (orange) nuclei. Shading shows standard deviation between embryo replicates. N = 5 nuclei per embryo, 3 embryo replicates for each surface. **(E)** Scatter plot showing nuclear volume and mean nuclear intensity. R^2^ is Pearson’s correlation. **(F)** Average number of hubs per nucleus in ventral (green) or lateral (orange) nuclei. **(G)** Scatter plot showing number of hubs per nucleus and mean nuclear intensity. R^2^ is Pearson’s correlation. **(H)** Cumulative probability of mean hub intensity for the start of the nc, “peak” where the nuclear intensity is highest during the cycle, and end of nc13 (left) and nc14 (right).
**Fig. S2. Design of MS2 reporter and Dorsal occupancy in enhancer regions. (A)** Design of *snail-MS2* from (44) along with Dorsal occupancy (Dorsal ChIP-nexus from (40)) at the endogenous enhancer region corresponding to the region in the reporter. **(B)** Design of *hunchback-MS2* from (44) along with Dorsal occupancy (Dorsal ChIP-nexus from (40)) at the endogenous enhancer region corresponding to the region in the reporter. **(C)** Design of *sog-MS2* from (45) along with Dorsal occupancy (Dorsal ChIP-nexus from (40)) at the endogenous enhancer region corresponding to the region in the reporter.
**Fig. S3. Dorsal hubs correlate with nuclear intensity and have a variety of dwell times. (A-C)** Scatterplots show correlation between mean Dorsal intensity **(A)**, overlapping hub volume **(B)** and overlapping hub intensity **(C)** at each gene locus. R^2^ is Pearson’s Correlation. **(D)** For each gene and nc, the cumulative probability of hub dwell time is fit to a three-component gaussian mixture model. The fits for each component and all three together are shown alongside the number of hubs (N).
**Fig. S4. Dorsal hub presence at target genes is related to transcriptional activation. (A)** For each representative MS2 intensity trace (colored line), the overlapping hub volume (black) and overlapping hub intensity (gray) is shown in time during nc14. **(B)** Each line chart from top to bottom shows the relative MS2 intensity, mean Dorsal intensity, overlapping hub intensity, or overlapping hub volume aligned to the first frame the MS2 spot is visible for each trace (time = 0 min). Shading shows standard deviation among replicate nuclei. N = 15 nuclei per gene per nc.
**Fig. S5. Dorsal hub stability and intensity influence burst kinetics. (A-D)** Representative traces for each gene in nc14 shown along with highlighting to show where each transcription burst is called. **(D-E)** Boxplots showing each quantified burst parameter (burst amplitude **(D)**, loading rate **(E)**, burst output **(F)**, burst duration **(G)**, and burst frequency **(H)**) if any hub is present at the start of the burst or not. For each boxplot, a Mann-Whitney U-test was performed to determine significance and following p-values were used: *p<0.05, **p<0.01, and ***p<0.001. **(I-M)** Scatter plots showing each burst parameter with the hub interaction durations after a burst **(I-M)** and mean Dorsal intensity before the burst **(N-R)** only when any hub is present at the start of the burst for ventrally imaged genes (left) and laterally imaged genes (right). A Pearson’s correlation R^2^ and linear regression is shown when p>0.05 for the correlation.

Supplement 2 **Movie 1**: Movies showing Dorsal-mNeonGreen (gray) in nuclei along the lateral (left) or ventral (right) surface of a *Drosophila* embryo in nc13 and nc14. Each movie is a 3D rendering with equivalent contrast limits.

Supplement 3 **Movie 2**: Movie showing Dorsal-mNeonGreen (gray) in 3D rendered nuclei along the lateral transition point of a *Drosophila* embryo in nc13 and nc14.

Supplement 4 **Movie 3**: Example of hub segmentation (colored labels) within ventral nuclei of Dorsal-mNeonGreen (gray). Movie starts on a single z-slice then moves through the whole nucleus in z.

Supplement 5 **Movie 4**: Example 3D rendering of nuclei through time showing Dorsal-mNeonGreen (gray) at MS2 spots (circled) for *sna, hb (v), sog,* and *hb (l)* in nc13.

Supplement 6 **Movie 5**: Example 3D rendering of nuclei through time showing Dorsal-mNeonGreen (gray) at MS2 spots (circled) for *sna, hb (v), sog,* and *hb (l)* in nc14. For visualization purposes, cytoplasm has been removed and set to zero.

Supplement 7 **Movie 6**: Example of 3D MS2 tracking in time within a single nucleus. Gray channel shows MCP-mCherry distribution in the nucleus. For visualization purposes, cytoplasm has been removed and set to zero. A pink circle marks the spot of nascent transcription of *sog*-MS2. The left shows a top-down (X-Y) view while the right shows a side (Y-Z) view.

## Figures and Tables

**Key Resources:** 

Reagent or Resource	Source	Identifier	Additional Information
**Fly Lines**			
Dorsal-mNeonGreen	Unpublished/this work	-	
MCP-mCherry	Gift from Garcia Lab	-	From (44)
*snail*-MS2	Gift from Garcia Lab	-	From (44)
*hunchback*-MS2	Gift from Garcia Lab	-	From (44)
*sog*-MS2	Gift from Lim Lab	-	From (45)
**Critical Commercial Reagents**			
Tetraspeck beads	Thermofisher	T7280	Beads for microscope calibration
Alexafluor dyes	Thermofisher	Alexa Fluor 488 Dye, Alexa Fluor 594 Dye	Dye for microscope calibration
Halocarbon Oil 27	Sigma-Aldrich	H8773-100 ML	
n-Heptane	McMaster	3190K548	
**Software**			
Custom image analysis kit for hub and MS2 interaction analysis	https://gitlab.com/mir-lab/publications/dorsal-ms-code-compilation-2025.git & doi: 10.5281/zenodo.15091745	N/A	Pre-processing for nuclear and hub segmentation adapted from (*18, 19*) for this paper. MS2 tracking and burst analysis created for this paper.
Imaris Parallel Writer	https://github.com/abcucberkeley/LLSM5DTools	N/A	From (46)
micro-SAM	https://github.com/computational-cell-analytics/micro-sam	N/A	From (47)
Cellpose 2.0	https://github.com/MouseLand/cellpose	N/A	From (48)

## Data Availability

All imaging data is available on request.

## Code availability.

Custom analysis software used in this study is available on Zenodo (10.5281/zenodo.15091745) and on Gitlab (https://gitlab.com/mir-lab/publications/dorsal-ms-code-compilation-2025.git).
