# Enhancer binding kinetics explain transcription factor hub formation

**DOI:** 10.1101/2025.04.07.647578

**Published:** 2026-07-21

**Authors:** Samantha Fallacaro, Manya Kapoor, Lillian Encarnation, Apratim Mukherjee, Meghan A. Turner, Hernan G. Garcia, Mustafa Mir

## Abstract

Transcription factors (TFs) form dynamic, high-concentration clusters, condensates, or hubs, proposed to increase TF binding frequency at target enhancers. However, how enhancer sequence shapes hub properties remains unclear. We developed a live-imaging based framework to quantify the spatiotemporal relationship between TF hubs and actively transcribed genes in live Drosophila embryos. Examining hubs formed by the TF Dorsal across enhancers with defined binding-site composition, we find that hub enrichment and persistence scale with the number of Dorsal binding motifs. However, these hub properties do not predict transcriptional bursts for a given enhancer. Combining quantitative imaging with computational modeling, we show that Dorsal hub formation can be explained by TF–DNA binding kinetics alone. These findings support a model in which TF hubs emerge from enhancer-encoded TF–DNA interactions rather than higher-order regulatory assemblies.

## Full Text Availability

The license terms selected by the author(s) for this preprint version do not permit archiving in PMC. The full text is available from the preprint server.

