# Supplementary material for: Transcription factor hubs exhibit gene-specific properties that tune expression
Source: bioRxiv. 2025 Apr 8:2025.04.07.647578. Preprint. [Version 1] doi: 10.1101/2025.04.07.647578 (PMC12026892; doi:10.1101/2025.04.07.647578)
Supplement: Supplement 1 — Fig. S1. Nuclear segmentation and hub detection across the dorsoventral axis. (A, B) Images showing where nuclei were selected at the lateral surface within a max projection of a full field of view (A) and for a single slice for each nuclei (B). Nuclei labelled as lateral (for sog and hunchback_l) were imaged within 5 nuclear rows of the transition point (row 0 of nuclei where the cytoplasm and nuclear signals are indistinguishable). Each row has a varying nuclear to cytoplasmic ratio of signal yet all have hubs within the nucleus. Scale bar in B is 15 microns and 1 micron in C. (C) Images showing results of nuclear and hub segmentation. Left image shows a representative 3D rendering of Dorsal-mNeonGreen in ventral nuclei. Scale bar is 2 microns. Center image shows nuclear segmentation of the 3D rendering. Right image shows segmentation of Dorsal hubs after applying a custom segmentation code. (D) Average nuclear volume in ventral (green) or lateral (orange) nuclei. Shading shows standard deviation between embryo replicates. N = 5 nuclei per embryo, 3 embryo replicates for each surface. (E) Scatter plot showing nuclear volume and mean nuclear intensity. R2 is Pearson’s correlation. (F) Average number of hubs per nucleus in ventral (green) or lateral (orange) nuclei. (G) Scatter plot showing number of hubs per nucleus and mean nuclear intensity. R2 is Pearson’s correlation. (H) Cumulative probability of mean hub intensity for the start of the nc, “peak” where the nuclear intensity is highest during the cycle, and end of nc13 (left) and nc14 (right). Fig. S2. Design of MS2 reporter and Dorsal occupancy in enhancer regions. (A) Design of snail-MS2 from (44) along with Dorsal occupancy (Dorsal ChIP-nexus from (40)) at the endogenous enhancer region corresponding to the region in the reporter. (B) Design of hunchback-MS2 from (44) along with Dorsal occupancy (Dorsal ChIP-nexus from (40)) at the endogenous enhancer region corresponding to the region in the reporter. (C) D [file media-1.pdf]

Supplementary Figures

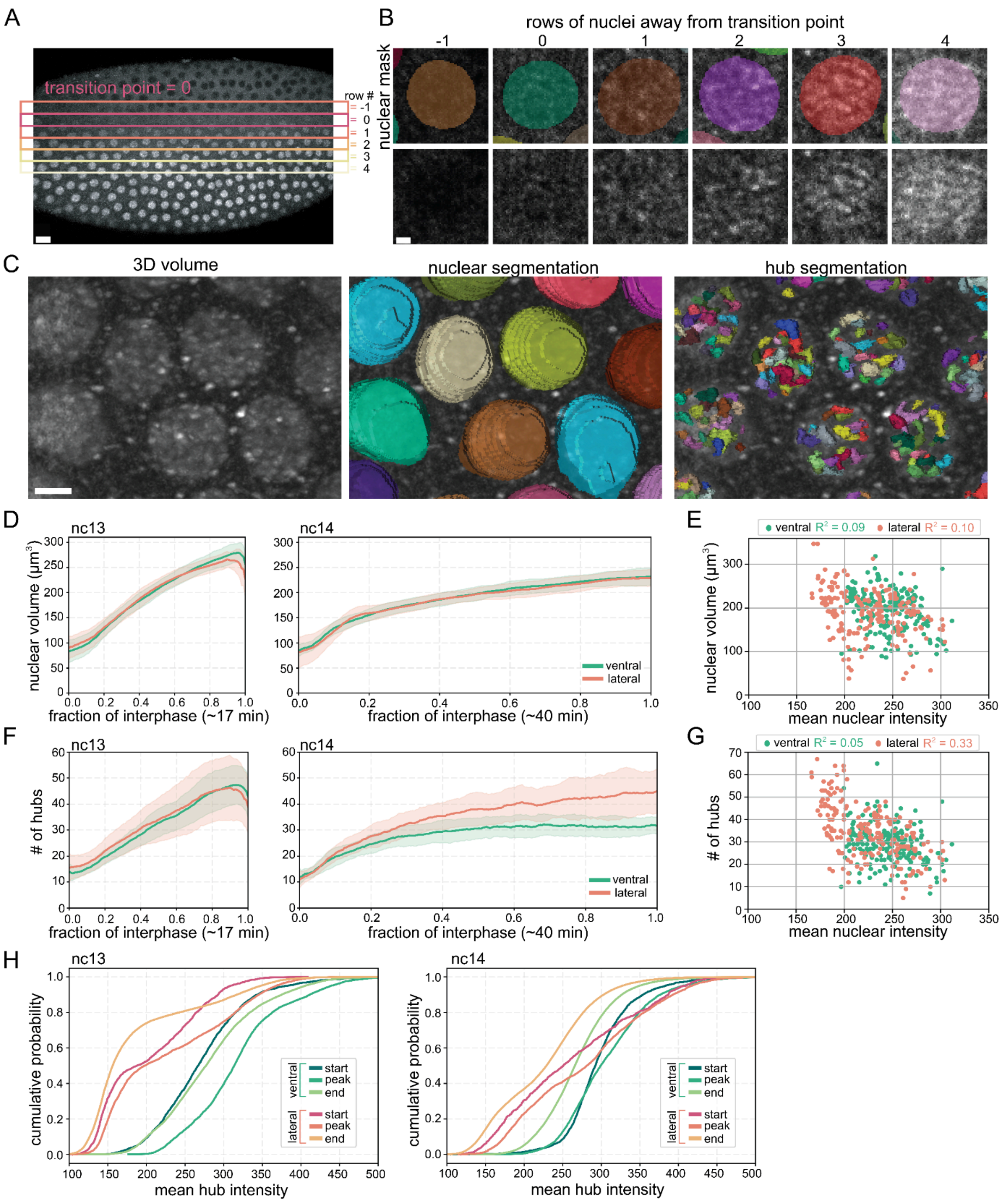

Fig. S1. Nuclear segmentation and hub detection across the dorsoventral axis. (A, B) Images showing where nuclei were selected at the lateral surface within a max projection of a full field of view **(A)** and for a single slice for each nuclei **(B)**. Nuclei labelled as lateral (for *sog* and *hunchback_l*) were imaged within 5 nuclear rows of the transition point (row 0 of nuclei where the cytoplasm and nuclear signals are indistinguishable). Each row has a varying nuclear to cytoplasmic ratio of signal yet all have hubs within the nucleus. Scale bar in B is 15 microns and 1 micron in C. **(C)** Images showing results of nuclear and hub segmentation. Left image shows a representative 3D rendering of Dorsal-mNeonGreen in ventral nuclei. Scale bar is 2 microns. Center image shows nuclear segmentation of the 3D rendering. Right image shows segmentation of Dorsal hubs after applying a custom segmentation code. **(D)** Average nuclear volume in ventral (green) or lateral (orange) nuclei. Shading shows standard deviation between embryo replicates. N = 5 nuclei per embryo, 3 embryo replicates for each surface. **(E)** Scatter plot showing nuclear volume and mean nuclear intensity. R^2 is Pearson's correlation. **(F)** Average number of hubs per nucleus in ventral (green) or lateral (orange) nuclei. **(G)** Scatter plot showing number of hubs per nucleus and mean nuclear intensity. R^2 is Pearson's correlation. **(H)** Cumulative probability of mean hub intensity for the start of the nc, "peak" where the nuclear intensity is highest during the cycle, and end of nc13 (left) and nc14 (right).

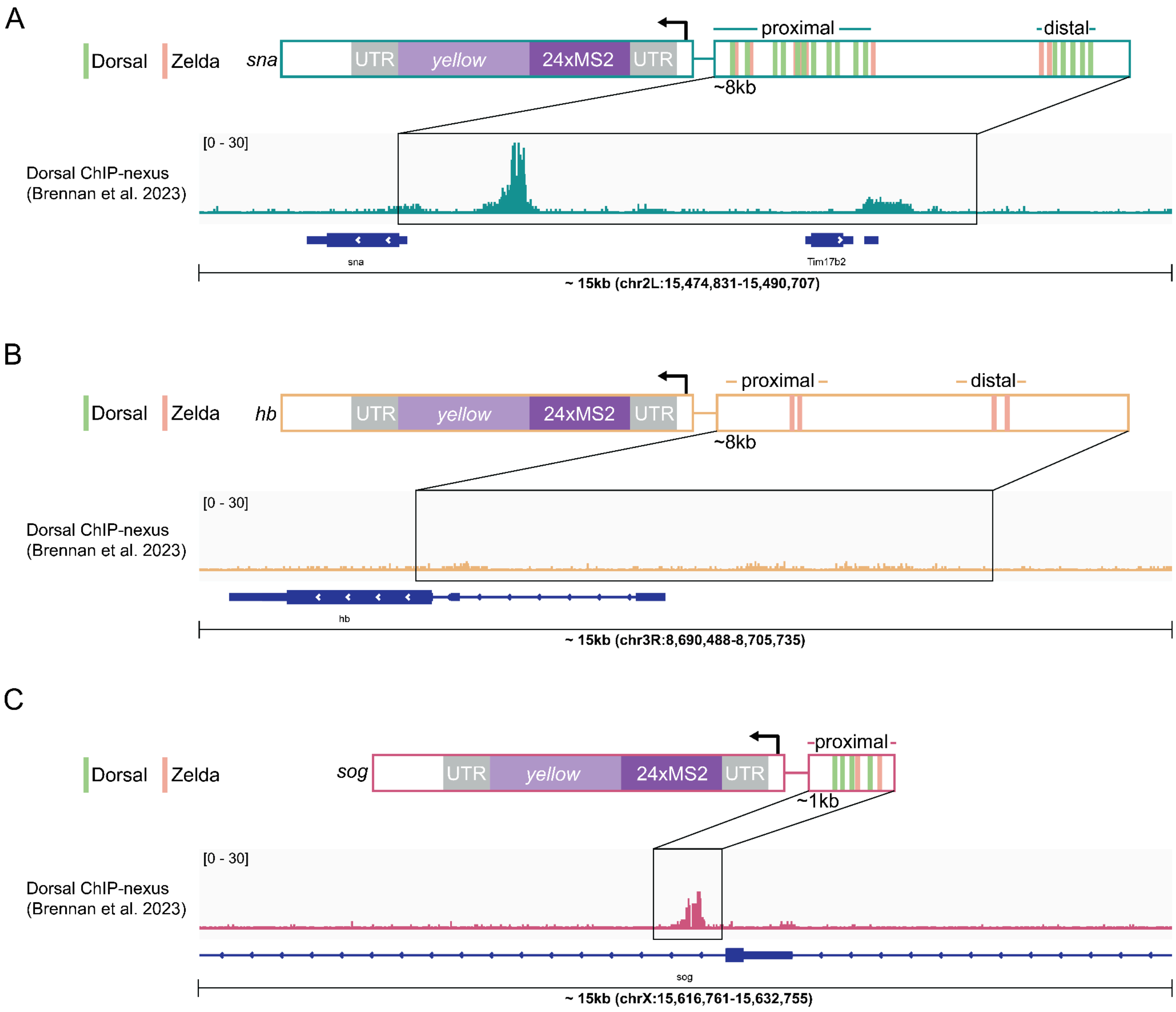

Fig. S2. Design of MS2 reporter and Dorsal occupancy in enhancer regions. (A) Design of *snail-MS2* from (*44*) along with Dorsal occupancy (Dorsal ChIP-nexus from (*40*)) at the endogenous enhancer region corresponding to the region in the reporter. **(B)** Design of *hunchback-MS2* from (*44*) along with Dorsal occupancy (Dorsal ChIP-nexus from (*40*)) at the endogenous enhancer region corresponding to the region in the reporter. **(C)** Design of *sog-MS2* from (*45*) along with Dorsal occupancy (Dorsal ChIP-nexus from (*40*)) at the endogenous enhancer region corresponding to the region in the reporter.

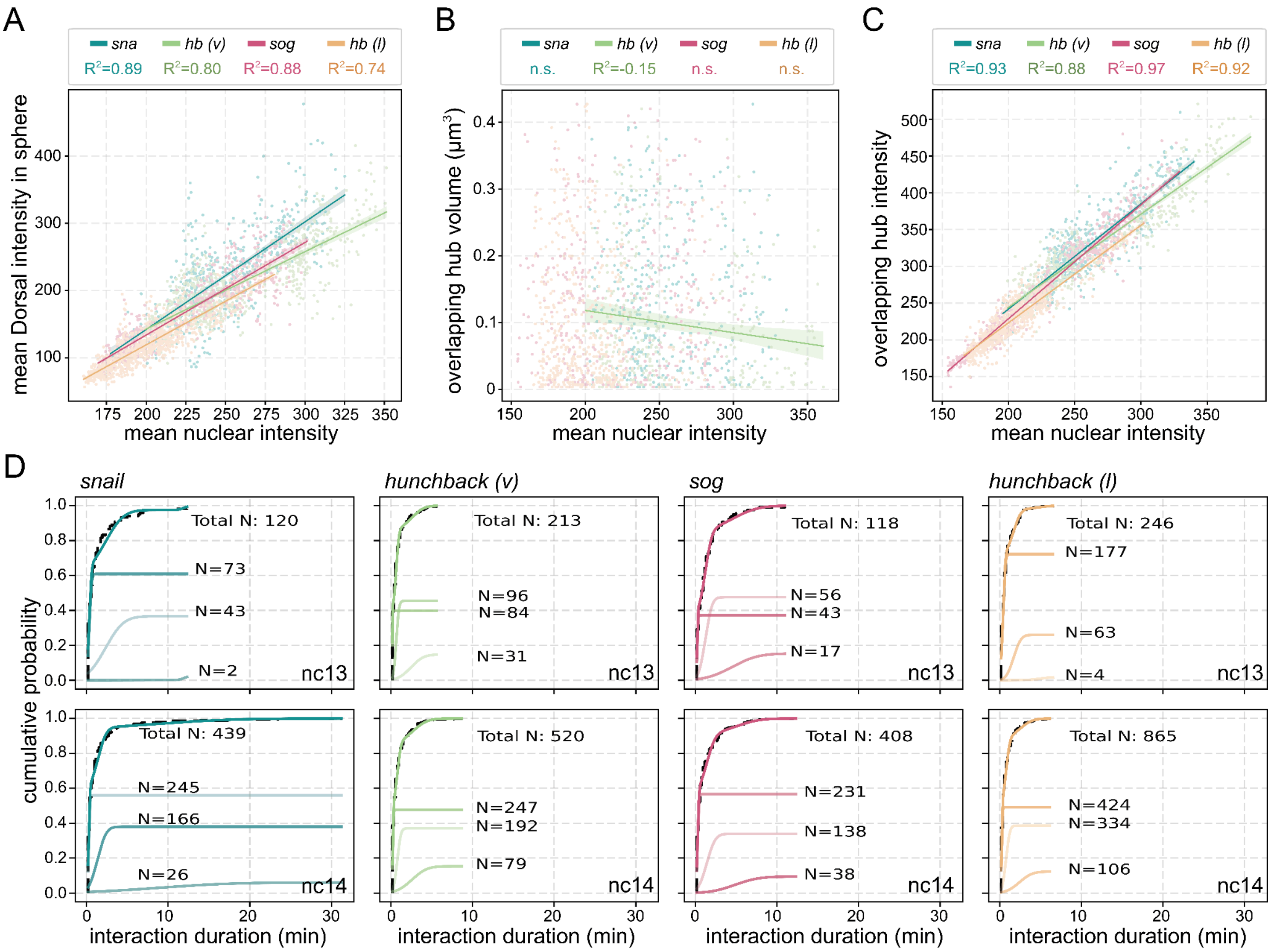

Fig. S3. Dorsal hubs correlate with nuclear intensity and have a variety of dwell times. (A-C) Scatterplots show correlation between mean Dorsal intensity **(A)**, overlapping hub volume **(B)** and overlapping hub intensity **(C)** at each gene locus. R^2 is Pearson's Correlation. **(D)** For each gene and nc, the cumulative probability of hub dwell time is fit to a three-component gaussian mixture model. The fits for each component and all three together are shown alongside the number of hubs (N).

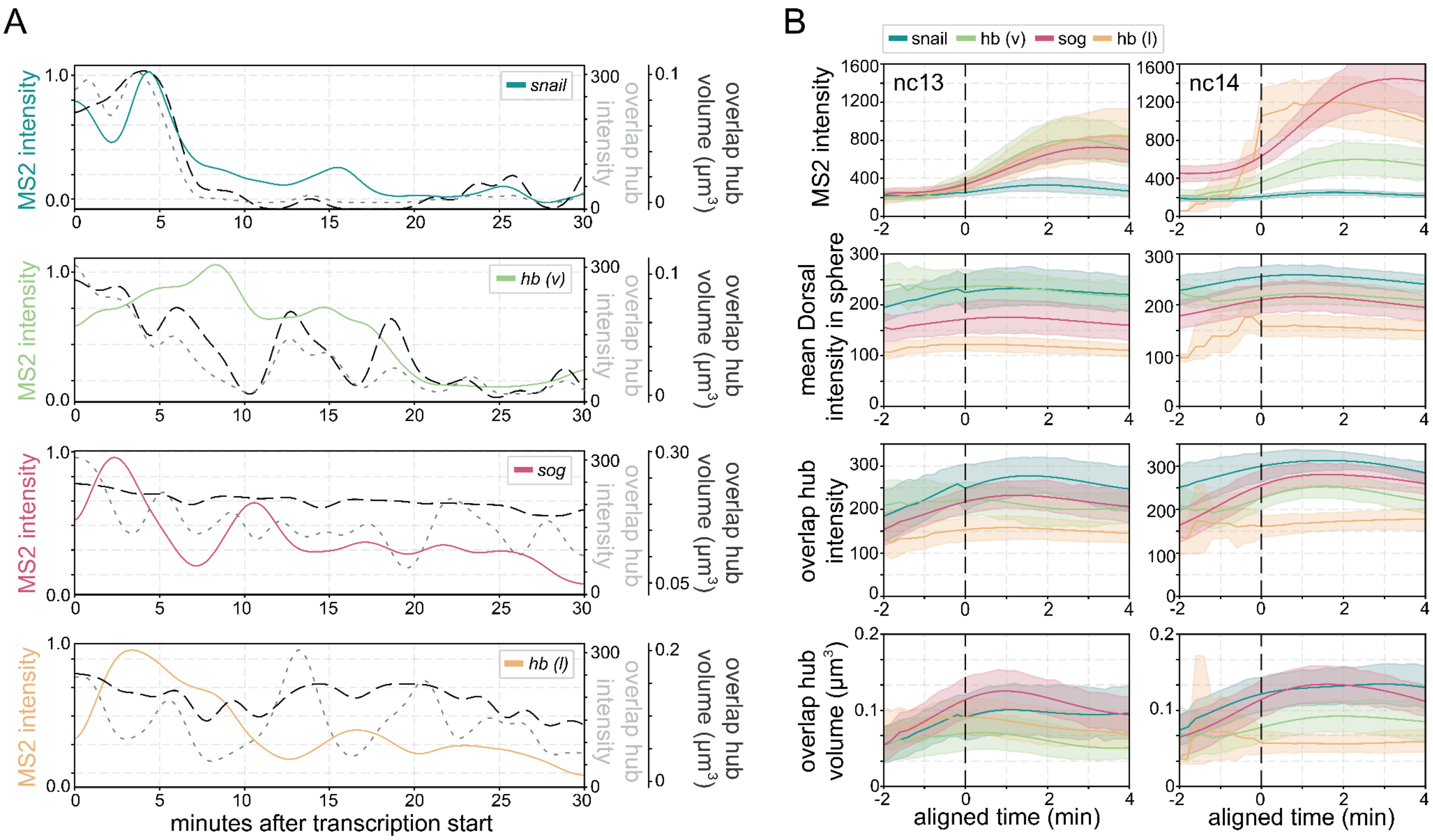

Fig. S4. Dorsal hub presence at target genes is related to transcriptional activation. (A) For each representative MS2 intensity trace (colored line), the overlapping hub volume (black) and overlapping hub intensity (gray) is shown in time during nc14. **(B)** Each line chart from top to bottom shows the relative MS2 intensity, mean Dorsal intensity, overlapping hub intensity, or overlapping hub volume aligned to the first frame the MS2 spot is visible for each trace (time = 0 min). Shading shows standard deviation among replicate nuclei. N = 15 nuclei per gene per nc.

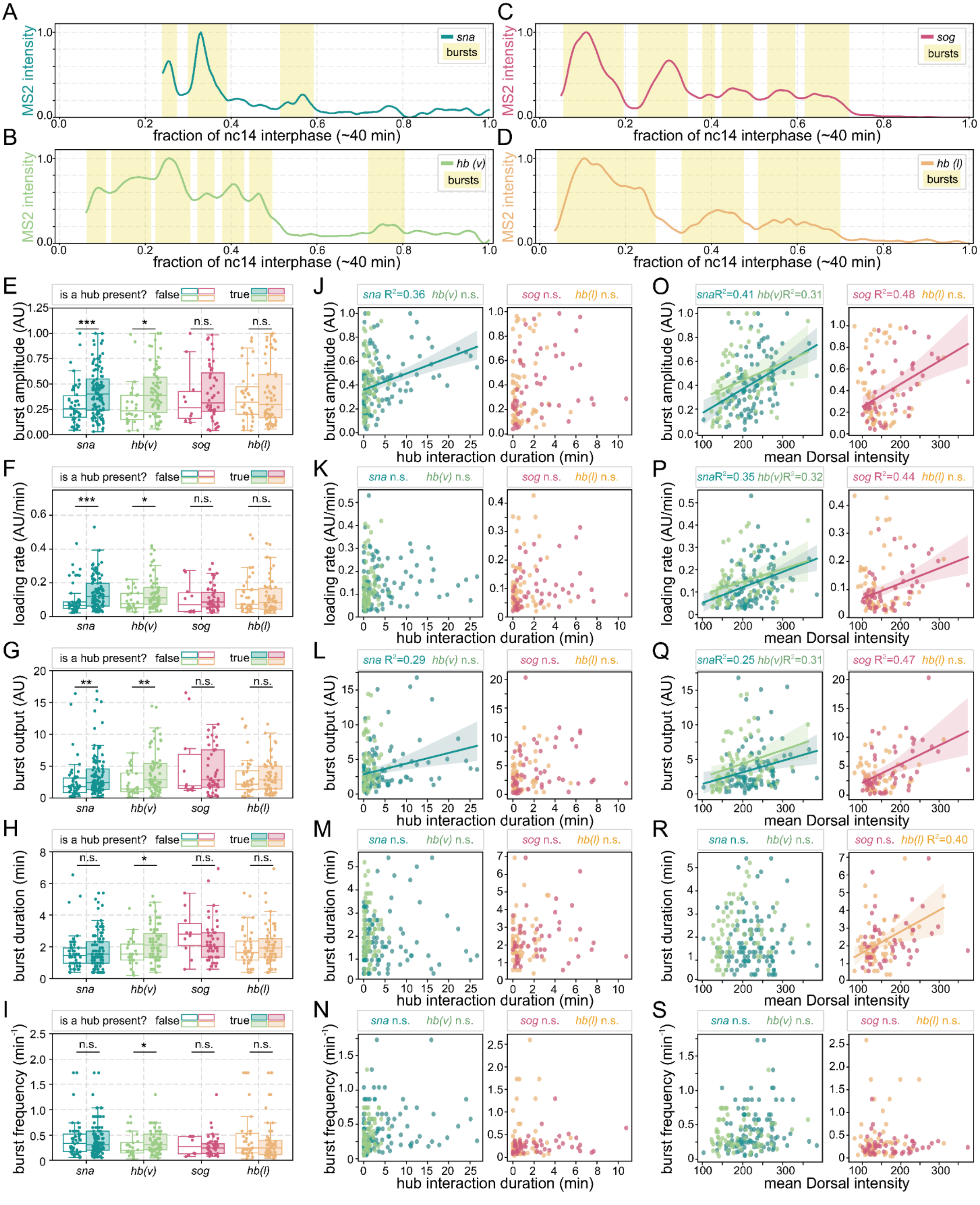

Fig. S5. Dorsal hub stability and intensity influence burst kinetics. (A-D) Representative traces for each gene in nc14 shown along with highlighting to show where each transcription burst is called. **(D-E)** Boxplots showing each quantified burst parameter (burst amplitude **(D),** loading rate **(E)**, burst output **(F)**, burst duration

(G), and burst frequency **(H)**) if any hub is present at the start of the burst or not. For each boxplot, a Mann-Whitney U-test was performed to determine significance and following p-values were used: *$p<0.05$, **$p<0.01$, and ***$p<0.001$. **(I-M)** Scatter plots showing each burst parameter with the hub interaction durations after a burst **(I-M)** and mean Dorsal intensity before the burst **(N-R)** only when any hub is present at the start of the burst for ventrally imaged genes (left) and laterally imaged genes (right). A Pearson's correlation R^2 and linear regression is shown when $p>0.05$ for the correlation.
